# Magnetic characterization of superparamagnetic nanoparticles pulled through model membranes

**DOI:** 10.1186/1477-044X-5-1

**Published:** 2007-01-04

**Authors:** Allison L Barnes, Ronald A Wassel, Fadee Mondalek, Kejian Chen, Kenneth J Dormer, Richard D Kopke

**Affiliations:** 1 Department of Physiology, College of Medicine, University of Oklahoma Health Sciences Center, 940 S.L. Young Blvd., Oklahoma City, OK 73104-0505, USA; 2 Hough Ear Institute, 3400 N.W. 56^th ^Street, Oklahoma City, OK 73112, USA; 3 School of Chemical, Biological & Materials Engineering, University of Oklahoma 100 East Boyd EC, Norman, OK 73019, USA

## Abstract

**Background:**

To quantitatively compare *in-vitro *and *in vivo *membrane transport studies of targeted delivery, one needs characterization of the magnetically-induced mobility of superparamagnetic iron oxide nanoparticles (SPION). Flux densities, gradients, and nanoparticle properties were measured in order to quantify the magnetic force on the SPION in both an artificial cochlear round window membrane (RWM) model and the guinea pig RWM.

**Methods:**

Three-dimensional maps were created for flux density and magnetic gradient produced by a 24-well casing of 4.1 kilo-Gauss neodymium-iron-boron (NdFeB) disc magnets. The casing was used to pull SPION through a three-layer cell culture RWM model. Similar maps were created for a 4 inch (10.16 cm) cube 48 MGOe NdFeB magnet used to pull polymeric-nanoparticles through the RWM of anesthetized guinea pigs. Other parameters needed to compute magnetic force were nanoparticle and polymer properties, including average radius, density, magnetic susceptibility, and volume fraction of magnetite.

**Results:**

A minimum force of 5.04 × 10^-16 ^N was determined to adequately pull nanoparticles through the in-vitro model. For the guinea pig RWM, the magnetic force on the polymeric nanoparticles was 9.69 × 10^-20 ^N. Electron microscopy confirmed the movement of the particles through both RWM models.

**Conclusion:**

As prospective carriers of therapeutic substances, polymers containing superparamagnetic iron oxide nanoparticles were succesfully pulled through the live RWM. The force required to achieve *in vivo *transport was significantly lower than that required to pull nanoparticles through the *in-vitro *RWM model. Indeed very little force was required to accomplish measurable delivery of polymeric-SPION composite nanoparticles across the RWM, suggesting that therapeutic delivery to the inner ear by SPION is feasible.

## Background

The use of superparamagnetic iron oxide nanoparticles (SPION) for the delivery of therapeutic molecules holds potential clinical applications, for it could provide substantial improvement over current techniques for drug delivery and gene transfection. Nanoparticles carrying therapeutic payloads could be targeted to a specific site, through directional acceleration by an external magnetic field. This field would pull the SPION to the target organ or tissue, where the biodegradable vehicles would subsequently break down, releasing drugs, DNA plasmids or bioactive molecules into surrounding tissues. An advantage that targeted delivery would have over systemic delivery is marked reduction in adverse side effects, which often decreases patient compliance. Another result of targeted delivery is that smaller dose (and cost) is required to achieve the same, or even improved, result. Others found in a rat model that with magnetic nanoparticles indomethacin had 60-fold higher concentrations and considerably reduced drug concentration in non-target organs [1]. Furthermore, magnetically susceptible nanoparticles, controlled by an external magnetic field, have the ability to reach target tissues that are difficult to access, such as the inner ear. Though these benefits are attractive, little progress has been made towards the goal of using SPION as *in-vivo *carriers of therapeutic payloads.

SPION are currently being used for cell separation [2], flow cytometry, immunoassays [3], and cellular labeling. One current in-vivo application of cellular labeling was made by derivatizing the nanoparticle with an HIV-TAT (Trans-Activating Transduction) peptide for promoting cellular internalization [4]. The HIV-TAT study not only demonstrated absence of cytotoxic effects or interference with cell function, but also took advantage of the property of SPION as contrast enhancing agents for magnetic resonance imaging (MRI). This may prove valuable in both clinical applications and future ferrite nanoparticle research as a method of imaging quantification. In addition, the utilization of the TAT cell penetrating peptides may be important for targeted delivery and gene transfection of cells that are non-dividing.

A recent study has shown for the first time, in a mammal, that adenoviral transfection with the MATH-1 gene can be used to regenerate inner ear hair cells and restore hearing [5]. MATH-1 expression within supporting cells along the Organ of Corti in the deafened cochlea, transformed these non-sensory cells into functional hair cells, and improved hearing thresholds. Nevertheless, safety limitations of adenoviruses used for gene therapy will likely deter use of this vector in clinical applications. Hence, a non-viral vector is currently being sought and preliminary studies are underway to incorporate MATH-1 plasmid DNA into a biocompatible polymeric-SPION for magnetic targeted delivery to the cochlea [6]. This technique would overcome the immune response complications and mutation risks that are involved in viral transfection [7]. Biodegradable poly-lactide co-glycolic acid (PLGA) polymers are attractive as carriers, because of their hydrophilicity, biocompatibility, promotion of cell membrane endocytosis and relative ease of derivitization with functional groups attached on the inside or outside of the polymer [8]. Programmed degradation of the polymer could result in timely, quantitative delivery of a drug, plasmid or other bioactive molecule.

Computing the magnetically induced mobility of SPION used as therapeutic carriers is important for their clinical applications. Magnetic force calculations aid in the determination of minimum field and magnetic material formulation, both to reduce technology and drug costs, and ensure patient safety. Force characterization is also a step towards determining the velocity of the particles through tissues, which is vital to developing dosing regimens. The equations for these two quantities are [9]:

Fm=HdHdyχfmV     (1) Force
 MathType@MTEF@5@5@+=feaafiart1ev1aaatCvAUfKttLearuWrP9MDH5MBPbIqV92AaeXatLxBI9gBaebbnrfifHhDYfgasaacH8akY=wiFfYdH8Gipec8Eeeu0xXdbba9frFj0=OqFfea0dXdd9vqai=hGuQ8kuc9pgc9s8qqaq=dirpe0xb9q8qiLsFr0=vr0=vr0dc8meaabaqaciaacaGaaeqabaqabeGadaaakeaacqWGgbGrdaWgaaWcbaGaemyBa0gabeaakiabg2da9iabdIeainaalaaabaGaemizaqMaemisaGeabaGaemizaqMaemyEaKhaaGGaciab=D8aJjabdAgaMnaaBaaaleaacqWGTbqBaeqaaOGaemOvayLaaCzcaiaaxMaadaqadaqaaiabigdaXaGaayjkaiaawMcaaiabbccaGGqabiab+zeagjab+9gaVjab+jhaYjab+ngaJjab+vgaLbaa@47A0@

v=2r2fmχμ0HdHdy9η     (2)Velocity
 MathType@MTEF@5@5@+=feaafiart1ev1aaatCvAUfKttLearuWrP9MDH5MBPbIqV92AaeXatLxBI9gBaebbnrfifHhDYfgasaacH8akY=wiFfYdH8Gipec8Eeeu0xXdbba9frFj0=OqFfea0dXdd9vqai=hGuQ8kuc9pgc9s8qqaq=dirpe0xb9q8qiLsFr0=vr0=vr0dc8meaabaqaciaacaGaaeqabaqabeGadaaakeaacqWG2bGDcqGH9aqpdaWcaaqaaiabikdaYiabdkhaYnaaCaaaleqabaGaeGOmaidaaOGaemOzay2aaSbaaSqaaiabd2gaTbqabaacciGccqWFhpWycqWF8oqBdaWgaaWcbaGaeGimaadabeaakiabdIeainaalaaabaGaemizaqMaemisaGeabaGaemizaqMaemyEaKhaaaqaaiabiMda5iab=D7aObaacaWLjaGaaCzcamaabmaabaGaeGOmaidacaGLOaGaayzkaaacbeGae4hiaaIae4NvayLae4xzauMae4hBaWMae43Ba8Mae43yamMae4xAaKMae4hDaqNae4xEaKhaaa@5291@

Thus, the magnetic force *F*_*m *_from an external permanent or electromagnet that would be used to pull SPION towards its pole face is dependent upon the field strength *H *and gradient *dH/dy *produced by the magnet. This equation can be rewritten in terms of the flux density *B *and simplified if one is using the CGS unit system, in which free space permeability μ_0 _is unity and where *B *is given in Gauss and *dB/dy *is in Gauss/cm.

Fm=BdBdyχfmV     (3) Force
 MathType@MTEF@5@5@+=feaafiart1ev1aaatCvAUfKttLearuWrP9MDH5MBPbIqV92AaeXatLxBI9gBaebbnrfifHhDYfgasaacH8akY=wiFfYdH8Gipec8Eeeu0xXdbba9frFj0=OqFfea0dXdd9vqai=hGuQ8kuc9pgc9s8qqaq=dirpe0xb9q8qiLsFr0=vr0=vr0dc8meaabaqaciaacaGaaeqabaqabeGadaaakeaacqWGgbGrdaWgaaWcbaGaemyBa0gabeaakiabg2da9iabdkeacnaalaaabaGaemizaqMaemOqaieabaGaemizaqMaemyEaKhaaGGaciab=D8aJjabdAgaMnaaBaaaleaacqWGTbqBaeqaaOGaemOvayLaaCzcaiaaxMaadaqadaqaaiabiodaZaGaayjkaiaawMcaaiabbccaGGqabiab+zeagjab+9gaVjab+jhaYjab+ngaJjab+vgaLbaa@478C@

The remaining factors are all properties of the SPION, which are the magnetic susceptibility χ (emu per Oe · cm^3^), the volume fraction of magnetite *f*_*m *_(dimensionless), and the overall particle volume *V *(cm^3^). The equation for velocity takes into consideration the drag force experienced by the particle moving through a substance of viscosity η according to Stokes' theorem. This involves determining the particle's radius *r*. The velocity equation can be further simplified to a function of *F*_*m *_and viscosity:

v=Fm6πrη     (4) Velocity
 MathType@MTEF@5@5@+=feaafiart1ev1aaatCvAUfKttLearuWrP9MDH5MBPbIqV92AaeXatLxBI9gBaebbnrfifHhDYfgasaacH8akY=wiFfYdH8Gipec8Eeeu0xXdbba9frFj0=OqFfea0dXdd9vqai=hGuQ8kuc9pgc9s8qqaq=dirpe0xb9q8qiLsFr0=vr0=vr0dc8meaabaqaciaacaGaaeqabaqabeGadaaakeaacqWG2bGDcqGH9aqpdaWcaaqaaiabdAeagnaaBaaaleaacqWGTbqBaeqaaaGcbaGaeGOnaydcciGae8hWdaNaemOCaiNae83TdGgaaiaaxMaacaWLjaWaaeWaaeaacqaI0aanaiaawIcacaGLPaaacqqGGaaiieqacqGFwbGvcqGFLbqzcqGFSbaBcqGFVbWBcqGFJbWycqGFPbqAcqGF0baDcqGF5bqEaaa@4709@

## Methods

### RWM experiments

The mobility of SPION was calculated and compared in two different physiological studies, that involved magnetically accelerating the particles through two round window membrane (RWM) models. The first study utilized data from a RWM model made of Madin-Darby Canine Kidney (MDCK) cells. These cells were grown on small intestinal submucosa (SIS) membrane fitted in cell culture plate inserts [8]. A tripartite cell culture membrane consisting of an upper MDCK epithelial cell layer, middle SWISS 3T3 fibroblasts layer, and a third layer of MDCK cells. Construction emulated the three layers of the human RWM: outer epithelium, loose connective tissue, and inner epithelium. A solution of 10 nm diameter dextran-encased magnetite nanoparticles (Micromod GmbH nanomag^®^-D, Germany) contained 50–130 nm aggregates. These SPION were placed on the upper layer of the culture, each in its holder, each in a well of a 24 well culture dish. The culture dish was then placed over a plastic casing containing 24 individual 1/4" (0.635 cm) 4.1 kilo-Gauss neodymium-iron-boron (NdFeB) disc magnets (MagStar Technologies™, Culver City, CA). The magnets were centrally positioned to the bottom of the culture plate wells, resulting in an operating distance of about 0.4 cm from the bottom cell layer to each magnet's pole face. After one hour exposure to the magnetic gradients, the fluid below the inserts was examined by transmission electron microscopy (TEM, Hitachi H7600) and proven to contain SPION.

The second study was performed on the RWM in anesthetized guinea pigs. The magnetite SPION were precoated with oleic acid and encapsulated in PLGA co-polymer. The oleic acid acts as a surfactant to prevent agglomeration of the magnetite particles and also decreases the likelihood of Fe_3_O_4 _oxidation. The polymer provides cellular compatibility and payload carrying capacity. A customized 4 inch (10.16 cm) 48 MGOe NdFeB cube magnet (Integrated Magnetics™, Culver City, CA) was used in this study. A Hall effect gaussmeter used for measurements (Model 5080, Sypris, Orlando, FL).

An incision was made behind the pinna of the guinea pig and bone was removed to expose the middle ear cavity. The RWM niche was visualized under an operating microscope and 3 μl of a 10^12 ^particles per ml solution of the SPION-polymer composite were placed in the niche using a microsyringe. The animal's head was then placed on the magnet, so the ear opposite the experimental ear RWM was aligned with the center of the magnet. This made the operating distance 1 inch (2.54 cm) from the experimental RWM to the pole face of the magnet. A heat lamp was used to keep the animal warm during placement on the magnet. After 20 minutes exposure time, the guinea pig was removed from the magnet. The solution of SPION-polymer remaining on the niche was wicked off, and fresh solution was added. After another 20 minutes exposure to the magnet, the process was repeated for a third and final time. The remaining solution was again wicked off before a small hole was made in the base of the cochlea with a syringe to extract the cochlear perilymphatic fluid.

**Figure 1:**
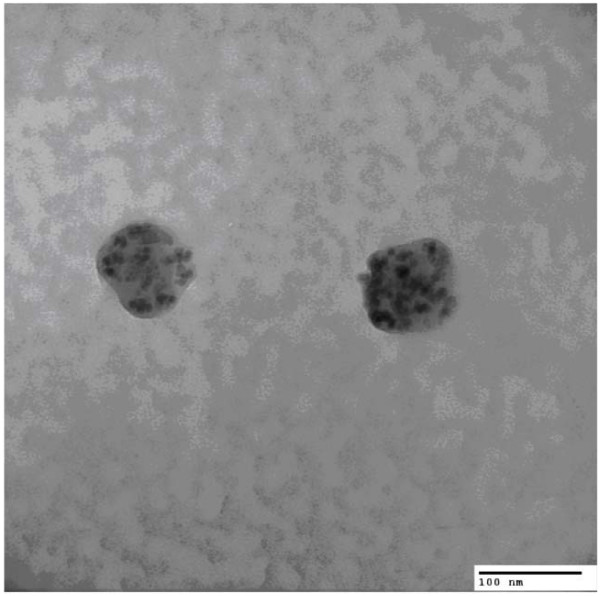
TEM of PLGA particles in experimental perilymph.

A control experiment was performed on the opposite ear, where all protocol remained the same, except no magnet was used. The guinea pig simply remained in the RWM surgical position for the 3 × 20 minute exposures. Perilymphatic fluids from both the experimental and control studies were aspirated into clean microsyringes, washed three times, and a TEM sample was taken from the bottom where the magnet had been used to concentrate the SPION polymer. TEM analysis demonstrated the presence of SPION-polymer nanoparticles in the perilymph for n = 2 trials (Figure 1). No SPION nanoparticles were found with TEM for the control experiments.

The success of both studies demonstrates two conditions in which sufficient forces were produced by different permanent magnets to pull SPION through both a viable RWM cell culture model and SPION-PLGA through a live RWM. The next step was to quantify the magnetic force that was present during these targeted delivery experiments.

### Magnetic field parameters

The Hall effect gaussmeter was used to measure the flux density over the surface of both magnets. To accomplish this, 1/4 inch (0.635 cm) grid paper was taped over the magnets, and the axial gaussmeter probe was positioned using a test tube holder at different heights above the magnet surface. These heights were selected to be centered around the operating distance from the magnet, to assist with calculation of the gradient. For the 24-well casing of disc magnets, the readings were taken at the surface (0 cm), 0.4 cm, and 0.8 cm. For the cube magnet, the heights were 0.5 inches (1.27 cm), 1 inch (2.54 cm), and 1.5 inches (3.81 cm).

Measurements were taken at each point on the grid paper to create a matrix of data points. For the 24-well casing, readings were only taken for a square surrounding the four centermost magnets. For the larger block magnet, measurements were taken at every other point on the grid. These data were plotted using graphics software (MatLab^® ^6.5) to create a three-dimensional flux density map. Spreadsheet software (Excel^® ^2003) was used to fit a trendline through the flux density values for the three distances at which measurements were made. The slope of this line provided the flux density gradient for that point on the grid. This was repeated for every data point, yielding a matrix of flux density gradients that could also be plotted in MatLab. A more descriptive parameter is the product of the flux density and gradient at each point, which is, according to Eq. (3), an index of the magnetic force. Plots of these *force index *data show the point on the magnet with the greatest pull on the SPION or SPION-PLGA particles.

### Particle size, susceptibility, and magnetite content

The nanomag^®^-D SPION had an average radius of 42 ± 16 nm and a density of 2.5 g/cm^3^. Susceptibility curves were obtained to provide the specific magnetization *s *in emu/g at a particular magnetic field strength. This is related to the susceptibility through the following equation

χ=sHρ     (5) Susceptibility,
 MathType@MTEF@5@5@+=feaafiart1ev1aaatCvAUfKttLearuWrP9MDH5MBPbIqV92AaeXatLxBI9gBaebbnrfifHhDYfgasaacH8akY=wiFfYdH8Gipec8Eeeu0xXdbba9frFj0=OqFfea0dXdd9vqai=hGuQ8kuc9pgc9s8qqaq=dirpe0xb9q8qiLsFr0=vr0=vr0dc8meaabaqaciaacaGaaeqabaqabeGadaaakeaaiiGacqWFhpWycqGH9aqpdaWcaaqaaiabdohaZbqaaiabdIeaibaacqWFbpGCcaWLjaGaaCzcamaabmaabaGaeGynaudacaGLOaGaayzkaaGaeeiiaaccbeGae43uamLae4xDauNae43CamNae43yamMae4xzauMae4hCaaNae4hDaqNae4xAaKMae4NyaiMae4xAaKMae4hBaWMae4xAaKMae4hDaqNae4xEaKNae4hlaWcaaa@4C27@

where ρ is the particle density. The magnetite content of these particles was given as a weight fraction of 82%, and was easily converted to a volume fraction using the particle's density.

The SPION-PLGA composite nanoparticles used in the second study was synthesized, therefore its properties needed to be measured. The average SPION-PLGA particle radius was measured by TEM to be 45 nm. The PLGA density was found to be 1.22 g/cm^3^, a value obtained from a study using similar polymers [10]. To obtain the magnetic susceptibility for the polymer containing multiple magnetite SPION, samples were sent to the Department of Physics, University of Nebraska-Lincoln to be tested using a vibrating sample magnetometer (VSM), the output of which is a susceptibility curve. The volume fraction of magnetite in the composite particle was found by thermal decomposition studies performed at the School of Chemical, Biological, and Materials Engineering, University of Oklahoma. This technique yielded a weight percentage that was again converted to a volume fraction of 0.01.

### Guidelines for animal research

All procedures involving animals were approved by the University of Oklahoma Institutional Animal Care and Utilization Committee, Protocol 14–141. Dunkin Hartley strain of guinea pigs (*Caviaporcellus*) were anesthetized using ketamine 70 mg/kg mixed with xylazine 7 mg/kg, injected intraperitoneally. Supplemental doses, 20% anesthetic dose, were given as needed.

## Results

**Figure 2:**
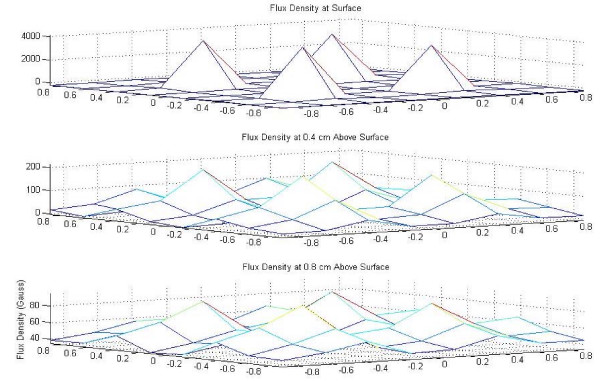
Flux density map for 24-well casing of NdFeB magnets, mesh plot.

The flux density plots created in MatLab^® ^for the 24-well magnet casing and cube magnet are shown in Figures 2 through 5, in both mesh and contour format. This data confirmed the assumption that the point of maximum flux density was the center of each of the disc magnets and the center of the entire block magnet.

**Figure 3:**
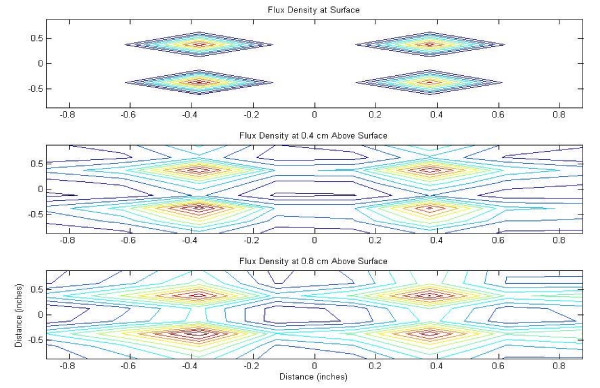
Flux density map for 24-well casing of NdFeB magnets, contour plot.

**Figure 4:**
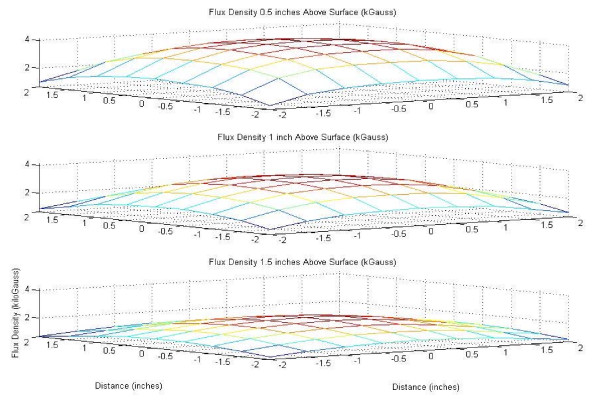
Flux density map for 4 inch 48 MGOe NdFeB magnet, mesh plot.

**Figure 5:**
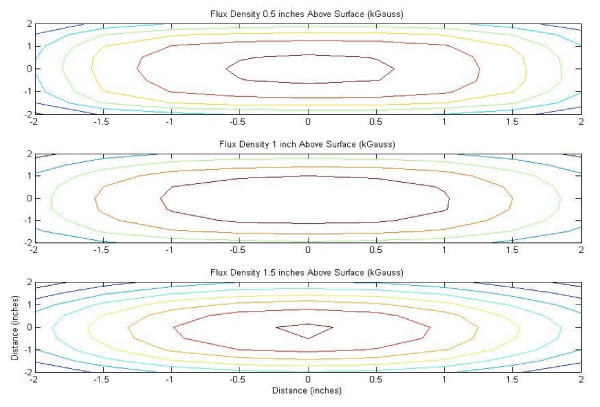
Flux density map for 4 inch 48 MGOe NdFeB magnet, contour plot.

**Figure 6:**
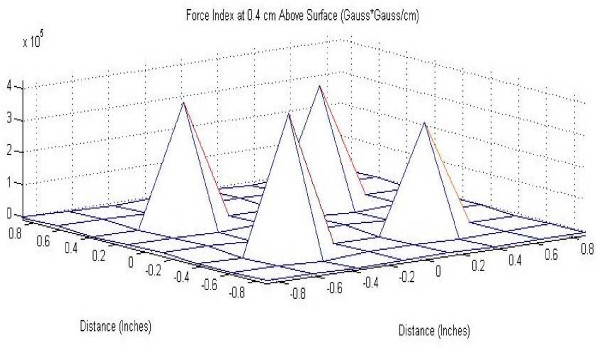
Force index produced by 24-well casing at 0.4 cm from magnet surface.

**Figure 7:**
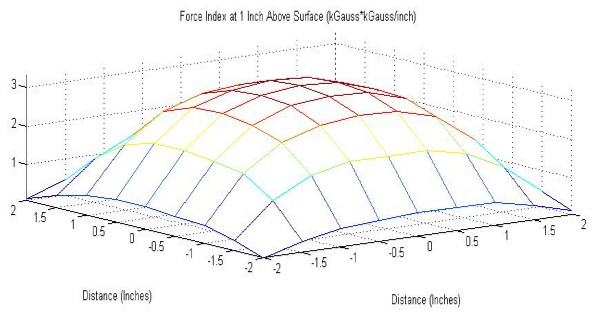
Force Index produced by block magnet at 2.54 cm from magnet surface.

Figures 6 and 7 show maps of the force index of each magnet studied. Note that the point of maximum force produced by the magnet is also the center, verifying the optimal position of the RWM during experiments.

The magnetic data collected for these two studies are provided in Table 1. The variability in flux density for each of the disc magnets is most likely due to the imprecise placement of the magnets in the plastic casing, as is the case with magnet 3, which protruded up from the well. The properties measured for the nanomag^®^-D and polymeric nanoparticles are provided in Table 2. By combining the data obtained in these two tables, force calculations were performed using Eq. (3). These results are summarized for each study in Table 3.

**Table 1:** Magnetic measurements determined by Hall Effect Gaussmeter.

	Flux Density at Operating Distance from RWM (Gauss)	Flux Density Gradient (Gauss/cm)
24-Well Casing of 40MGOe NdFeB Disc Magnets		
Magnet 1	198.6	1871
Magnet 2	198.2	1814.8
Magnet 3	210.6	2007.6
Magnet 4	184.9	1770.8
48MGOe NdFeB Block Magnet	3390	374.8

**Table 2:** Magnetite nanoparticle and PLGA polymeric nanoparticle properties.

	Magnetic susceptibility (emu/Oe · cm^3^)	Volume fraction of Fe_3_O_4_	Volume (cm^3^)
Nanomag^®^-D particles	0.285	0.47	1.15 × 10^-15^
Polymeric nanoparticles	0.002	0.01	3.81 × 10^-16^

**Table 3:** Force calculations for in-vitro and in-vivo studies.

	Magnetic Force (N)	
In-Vitro Study at 0.4 cm		
24-Well Casing of 40MGOe NdFeB Disc Magnets	Magnet 1	5.72392E-16
	Magnet 2	5.54081E-16
	Magnet 3	6.51293E-16
	Magnet 4	5.04367E-16
In-Vivo Study at 2.54 cm	48MGOe NdFeB Block Magnet	9.68176E-20

## Discussion

The nanomag^®^-D dextran encapsulated magnetite SPION showed much greater magnetic susceptibility (140 times) than the polymeric composite nanoparticles. This is expected due to the small amount of magnetite in the PLGA polymer and the very slightly paramagnetic nature of the PLGA itself. As a side investigation, the specific magnetization of the polymer alone was found using VSM to be 0.0038 emu/g at the same magnetic field strength of 3390 Oe. This corresponds by density to a very low magnetic susceptibility of 1.37 × 10^-6 ^emu/Oe · cm^3^. Still, these values correspond well with current data reported in literature 11, and it is important to note that even though the magnetic susceptibility of polymeric nanoparticles is very low, the polymer is crucial for delivery of a therapeutic payload.

The flux density produced by the block magnet at a distance of 2.54 cm from its surface is sixteen times that produced by the disc magnets at a distance of only 0.4 cm. This underlines the necessity of using such a high power magnet for *in-vivo *applications where the RWM is farther from the pole face of the magnet. The magnets used in the *in-vitro *study would not have produced sufficient flux density or gradient at the operating distances required for in-vivo delivery.

To determine the velocity of the nanoparticles used in these studies, the viscosity of the surrounding environment is needed. Since viscosity is typically a parameter used to describe liquids, it was not a property of the RWM that could be easily measured, and its value depends on the path taken by the SPION through the tissue. Superparamagnetic nanoparticles follow flux lines down a converging magnetic gradient; however, another factor is barriers within the tissue. Thus, nanoparticles will be accelerated toward the magnet, but may follow a pathway of low mechanical resistance, such as an intracellular pathway versus a tight junction. Previous studies have reported intracellular viscosity to be close to that of water, around 0.01 Poise [12]. However, the RWM consists not only of upper and lower confluent cellular layers, but also of loose collagen matrix of which viscosities are much higher, around 80 to 130 Poise [13]. Experiments are underway to determine the viscosity of the in-vitro RWM model in relation to a known gelatin viscosity.

For future in-vivo testing and clinical applications it will also be necessary to determine the velocity of the SPION or SPION-PLGA as they move within the cochlea in response to an external magnetic field. Perilymphatic fluid has a viscosity slightly lower than water, 0.0084 to 0.0087 Poise (measured at 20°C [14]). The predicted velocity in cochlear perilymphatic fluid (η = 0.00855 Poise) is given in Table 4 for both studies. Magnet 3 was selected for the in-vitro study because it would provide the maximum expected velocity.

**Table 4:** Predicted velocities in the cochlear duct.

	Particle Size (μm)	Velocity in Perilymph (cm/s)
In-Vitro Study (Magnet 3)	0.065	6.22036E-05
In-Vivo Study	0.045	9.24685E-09

The velocity found for the in-vitro study corresponds well with data found in a similar study using larger magnetic microspheres [15]. However, the velocity found in the in-vivo study is much smaller than expected for particles of this size, mainly due to the low susceptibility and volume fraction of magnetite. Predicted velocities through the RWM will be even lower than what was found for the perilymph, because of the higher viscosity of the tissue.

## Conclusion

This work comprises the first full characterization of polymeric superparamagnetic nanoparticle delivery through living tissue in a target organ. Although neither of these studies involved SPION containing a specific therapeutic payload, the successful movement of the *in-vitro *nanoparticles demonstrates sufficient magnetic force and desirable nanoparticle properties. The next step will involve loading the SPION-PLGA with a therapeutic biomolecule, looking for timed, quantifiable, targeted release of the biomolecule into the perilymph for access to inner ear supporting cells. This work outlines how future studies can be done to select external magnetic field requirements for specific nanoparticles to achieve a certain force and velocity. Conversely, a nanoparticle could be optimally designed for a given permanent magnet. For *in-vivo *testing, magnets with sufficient flux density and gradient at distances of at least 2.54 cm (rodent models) are required. Polymers need to be designed that have high magnetic susceptibility, either by increasing the size of the polymer itself or by increasing the magnetite content.

## Authors' contributions

AB designed, conducted and analyzed the magnetics experiments, participated in the animal experiments and drafted the manuscript. RW prepared, characterized (size, composition, volume fraction and magnetic susceptibility) and analyzed vibrating sample magnetometry and electron microscopy results. FM performed the cell culture magnetics experiments and data analysis. KC performed the animal experiments and analyzed the data. RK contributed to theround window in vitro and in vivo experimental designs and data analysis and electron microscopy analysis. KD participated in the design, conductance and oversight ofin vitro and in vivo experiments, writing and critique of the manuscript.

## Declaration of competing interests

The author(s) declare that they have no competing interests.

## References

[B1] Vyas SP, Malaiya A (1989). In vivo characterization of indomethacin magnetic polymethyl methacrylate nanoparticles. J Microencapsul.

[B2] Zhang H, Moore LR, Zborowski M, Williams PS, Margel S, Chalmers JJ (2005). Establishment and implications of a characterization method for magnetic nanoparticle using cell tracking velocimetry and magnetic susceptibility modified solutions. Analyst.

[B3] Lizard G, Monier S, Prunet C, Duvillard L, Gambert P (2004). [Microspheres, nanospheres and flow cytometry: from cellular to molecular analysis]. Ann Biol Clin (Paris).

[B4] Lewin M, Carlesso N, Tung CH, Tang XW, Cory D, Scadden DT, Weissleder R (2000). Tat peptide-derivatized magnetic nanoparticles allow in vivo tracking and recovery of progenitor cells. Nat Biotechnol.

[B5] Izumikawa M, Minoda R, Kawamoto K, Abrashkin KA, Swiderski DL, Dolan DF, Brough DE, Raphael Y (2005). Auditory hair cell replacement and hearing improvement by Atoh1 gene therapy in deaf mammals. Nat Med.

[B6] Kopke D, Wassel RA, Mondalek F, Howard E, Grady B, Chen K, Liu J, Gibson D, Dormer KJ (2006). Magnetic Nanoparticles: Inner Ear Targeted Molecule Delivery and Middle Ear Implant. Audiology & Neurotology.

[B7] Haddada H, Cordier L, Perricaudet M (1995). Gene therapy using adenovirus vectors. Curr Top Microbiol Immunol.

[B8] Mondalek FG, Zhang YY, Kropp B, Kopke R, Ge X, Jackson RL, Dormer KJ (2006). The permeability of SPION over an artificial three-layer membrane is enhanced by external magnetic field. J Nanobiotechnology.

[B9] Hafeli UO, Lobedann MA, Steingroewer J, Moore LR, Riffle J (2005). Optical method for measurement of magnetophoretic mobility of individual magnetic microspheres in defined magnetic field. J Magn Mag Mat.

[B10] Vauthier C, Schmidt C, Couvreur P (1999). Measurement of the density of polymeric nanoparticulate drug carriers by isopycnic centrifugation. Journal of Nanoparticle Research.

[B11] Harris LA, Goff JD, Carmichael AY, Riffle JS, Harburn JJ, St. Pierre TG, Saunders M (2003). Magnetite nanoparticle dispersions stabilized with triblock copolymers. Chem Mater.

[B12] Fushimi K, Verkman AS (1991). Low viscosity in the aqueous domain of cell cytoplasm measured by picosecond polarization microfluorimetry. J Cell Biol.

[B13] Chan RW, Titze IR (1998). Viscosities of implantable biomaterials in vocal fold augmentation surgery. Laryngoscope.

[B14] Schnieder EA, Schindler K (1968). Hexosamine in the inner ear fluids of man and guinea-pig. Arch Klin Exp Ohren Nasen Kehlkopfheilkd.

[B15] Hafeli UO, Ciocan R, Dailey JP (2002). Characterization of Magnetic Particles and Microspheres and Their Magnetophoretic Mobility Using a Digital Microscopy Method. 4th International Conference on the Scientific and Clinical Applications of Magnetic Carriers.

